# Infrared Spectroscopic Evidences of Strong Electronic Correlations in (Sr_1−*x*_La_*x*_)_3_Ir_2_O_7_

**DOI:** 10.1038/srep32632

**Published:** 2016-09-07

**Authors:** Gihyeon Ahn, S. J. Song, T. Hogan, S. D. Wilson, S. J. Moon

**Affiliations:** 1 Department of Physics, Hanyang University, Seoul 04763, Korea; 2 Department of Physics, Boston College, Chestnut Hill, Massachusetts 02467, USA; 3 Department of Materials, University of California, Santa Barbara, California 93106, USA

## Abstract

We report on infrared spectroscopic studies of the electronic response of the (Sr_1−*x*_La_*x*_)_3_Ir_2_O_7_ system. Our experiments revealed hallmarks of strong electronic correlations in the evolution of the electronic response across the filling-controlled insulator-metal transition. We observed a collapse of the *J*_eff_ = 1/2 Mott gap accompanying the transfer of the spectral weight from the high-energy region to the gap region with electron doping. The intraband conductivity at the metallic side of the transition was found to consist of coherent Drude-like and incoherent responses. The sum rule and the extended Drude model analyses further indicated a large mass enhancement. Our results demonstrate a critical role of the electronic correlations in the charge dynamics of the (Sr_1−*x*_La_*x*_)_3_Ir_2_O_7_ system.

Electronic correlations have been the source of numerous exotic phenomena including metal-insulator transitions and high-*T*_c_ superconductivity in 3*d* and 4*d* transition metal oxides^1^. A new frontier in the search for correlation-driven novel states is the exploration of 5*d* iridium oxides in which the energy scales of electron hopping, Coulomb interaction, and spin-orbit coupling are comparable. The members of the iridate Ruddlesden-Popper series Sr_*n*+1_Ir_*n*_O_3*n*+1_ are of particular interest. In the *n* = 1 and *n* = 2 members of the series, the electronic correlations in concert with the strong spin-orbit coupling were found to lead to the formation of an effective total angular momentum *J*_eff_ = 1/2 Mott state^2^,^3^,^4^,^5^,^6^,^7^. Subsequent theoretical and experimental studies on charge carrier doped Sr_2_IrO_4_ revealed the signatures of the pseudogap and *d*-wave superconductivity^8^,^9^,^10^,^11^,^12^,^13^,^14^,^15^, which implies an importance of electronic correlations in Sr_2_IrO_4_ system.

In contrast, the role of electronic correlations in bilayered Sr_3_Ir_2_O_7_ system is under intense debate. A neutron scattering experiments showed nearly identical ordered magnetic moments of Sr_3_Ir_2_O_7_ and Sr_2_IrO_4_, suggesting a similar strength of the electronic correlations in the two compounds^16^,^17^. A scanning tunneling spectroscopy (STS) experiment of Sr_3_Ir_2_O_7_ in combination with density-functional-theory (DFT) calculations suggested a critical role of electronic correlations in the formation of a substantial charge gap of about 130 meV^18^. The STS data further showed that the oxygen-vacancy-induced electronic structure changes were reminiscent of the filling-controlled Mott transitions in 3*d*/4*d* transition metal oxides. On the other hand, an angle-resolved photoemission spectroscopy (ARPES) experiment suggested a conventional Fermi-liquid state of (Sr_1 − *x*_La_*x*_)_3_Ir_2_O_7_ with a small mass renormalization of about 2^19^. Band structure calculations also suggested that Sr_3_Ir_2_O_7_ might be almost a band insulator^19^,^20^,^21^,^22^. A tight-binding calculation predicted that a direct gap between the bands mainly composed of the *J*_eff_ = 1/2 states occurred at every momentum point and the Fermi level was found to barely touch the bottom of the occupied bands and the top of the unoccupied bands. Electronic correlations merely pushed the bands away from the Fermi level leading to the insulating state^22^.

Our paper provides experimental evidences of strong electronic correlations in (Sr_1 − *x*_La_*x*_)_3_Ir_2_O_7_. We investigated the electronic response of (Sr_1−*x*_La_*x*_)_3_Ir_2_O_7_ by using infrared spectroscopy. We found that electron doping led to an insulator-metal transition that is reminiscent of the Mott transitions in strongly correlated 3*d*/4*d* transition metal oxides. We observed a spectral weight transfer from high energies to the ingap region to fill the charge gap of the parent compound. The intraband conductivity of the electron-doped compounds was found to be composed of a coherent and an incoherent responses. The sum rule and the extended Drude model analyses of our optical conductivity data revealed a substantial mass enhancement of the charge carriers in the metallic compound.

## Results and Discussion

**Figure 1:**
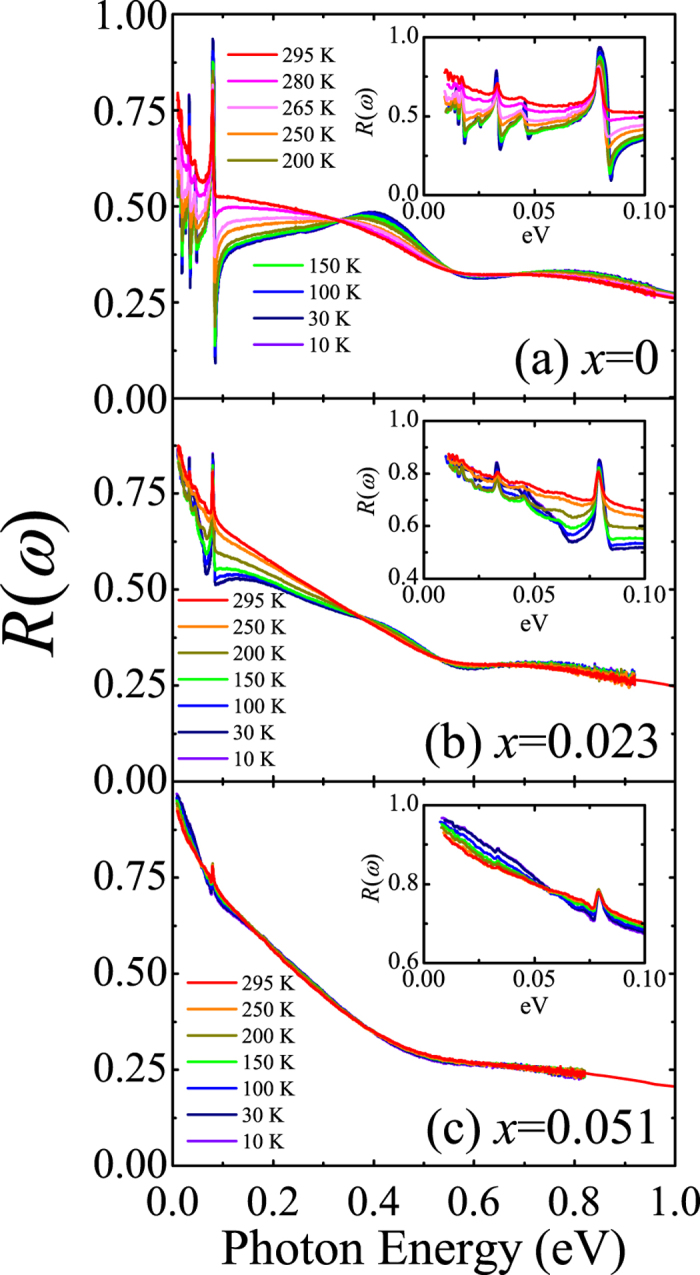
Temperature-dependent reflectivity spectra *R*(*ω*) of (**a**) Sr_3_Ir_2_O_7_ (*x* = 0), (**b**) (Sr_0.977_La_0.023_)_3_Ir_2_O_7_ (*x* = 0.023), and (**c**) (Sr_0.949_La_0.051_)_3_Ir_2_O_7_ (*x* = 0.051). Insets: *R*(*ω*) below 0.1 eV.

Figure 1 displays the reflectivity spectra *R*(*ω*) of (Sr_1−*x*_La_*x*_)_3_Ir_2_O_7_. The reflectivity data show that the electron doping induces an insulator-metal transition. Insulating behavior is evident in *R*(*ω*) of Sr_3_Ir_2_O_7_. At 10 K, the far-infrared reflectivity is dominated by sharp peaks which originate from infrared-active phonons as shown in Fig. 1(a). As temperature increases, *R*(*ω*) below 0.3 eV is enhanced accompanying a change in its slope; at higher temperatures *R*(*ω*) rises toward lower frequency implying the onset of an electronic contribution due to thermally-induced carriers^23^. Two broad peaks at about 0.4 and 0.8 eV represent the optical excitations between the *J*_eff_ bands, which will be discussed in detail later. Upon electron doping, the absolute magnitude of *R*(*ω*) below 0.3 eV is enhanced and the peak structure at about 0.4 eV is suppressed [Fig. 1(b,c)]. The spectral shape of the low-temperature *R*(*ω*) also changes with doping; *R*(*ω*) below 0.3 eV of the La-doped compounds increases toward lower frequency, which is in sharp contrast to the spectral shape of *R*(*ω*) of the parent compound. These observations indicate the existence of itinerant carriers in the doped materials.

**Figure 2:**
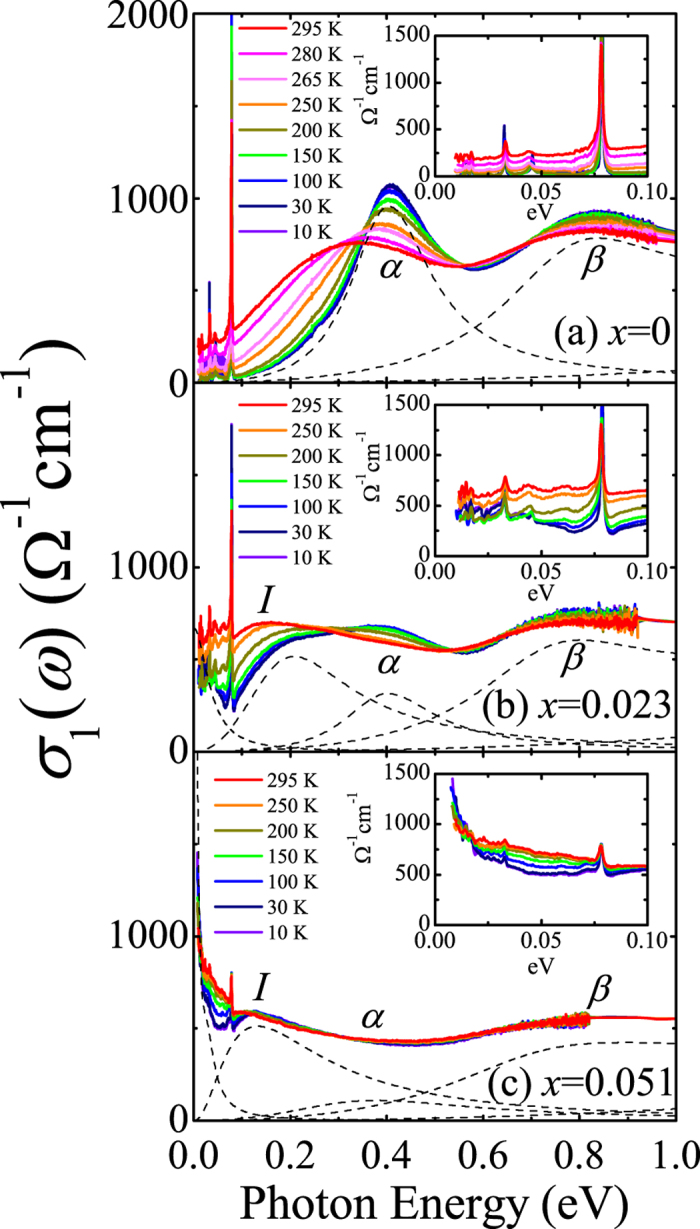
Real part of the optical conductivity spectra *σ*_1_(*ω*) of (**a**) Sr_3_Ir_2_O_7_ (*x* = 0), (**b**) (Sr_0.977_La_0.023_)_3_Ir_2_O_7_ (*x* = 0.023), and (**c**) (Sr_0.949_La_0.051_)_3_Ir_2_O_7_ (*x* = 0.051). Dashed lines represent the oscillators used to fit *σ*_1_(*ω*) at 10 K. We note that the oscillator labeled as *β* is composed of two Lorentzians. Insets: *σ*_1_(*ω*) below 0.1 eV.

Insight into the evolution of the electronic response with electron doping can be gained from the analysis of optical conductivity data. Figure 2 shows the real part of the optical conductivity spectra *σ*_1_(*ω*) of (Sr_1−*x*_La_*x*_)_3_Ir_2_O_7_. The conductivity data of the parent compound Sr_3_Ir_2_O_7_ can be interpreted in terms of the *J*_eff_ = 1/2 Mott state^4^. The two peaks labeled as *α* and *β* in Fig. 2(a) correspond to the optical transitions to the *J*_eff_ = 1/2 upper Hubbard band from the *J*_eff_ = 1/2 lower Hubbard band and from the *J*_eff_ = 3/2 band, respectively. The direct optical gap of Sr_3_Ir_2_O_7_ at 10 K is estimated to be about 0.3 eV. The optical gap decreases progressively with increasing temperature accompanying the spectral weight transfer from the energy region above 0.3 eV to lower energies. This temperature dependence was attributed to the enhancement of the phonon-assisted optical excitations^23^.

Electron doping prompts the collapse of the Mott gap. The optical transitions inherent to the *J*_eff_ = 1/2 Mott state, i.e., the peaks *α* and *β* are depressed upon electron doping. The spectral weight removed from the high energies (>0.3 eV) is shifted to the energy region below the optical gap of the parent compound thus filling the Mott gap. In (Sr_0.977_La_0.023_)_3_Ir_2_O_7_ the spectral weight forms a localized mode centered at about 0.18 eV along with a featureless conductivity below 0.1 eV, as shown in Fig. 2(b). This indicates an incoherent nature of the charge transport in (Sr_0.977_La_0.023_)_3_Ir_2_O_7_. The conductivity below about 0.05 eV increases slightly when the temperature is lowered across 150 K, which is consistent with the temperature dependence of the resistivity data^17^. We note that the temperature evolution of *σ*_1_(*ω*) of (Sr_0.977_La_0.023_)_3_Ir_2_O_7_, i.e., the enhancement of the conductivity below 0.3 eV with the spectral weight transfer from high to low energies, is essentially the same as that of the parent compound.

Further electron doping leads to the development of a coherent electronic response at the expense of the peaks *α* and *β*. The conductivity data of (Sr_0.949_La_0.051_)_3_Ir_2_O_7_ shown in Fig. 2(c) clearly exhibit a Drude-like peak centered at *ω* = 0 eV. The Drude-like response becomes narrower with the lowering of temperature, resulting in the enhancement of the conductivity at the lowest energies continued to the DC value. This temperature dependence is qualitatively different from those of *σ*_1_(*ω*) of Sr_3_Ir_2_O_7_ and (Sr_0.977_La_0.023_)_3_Ir_2_O_7_. In the latter compounds, the conductivity at the lowest energies is suppressed with decreasing temperature. The spectral shape and the temperature dependence of *σ*_1_(*ω*) thus clearly demonstrate a coherent metallic character of (Sr_0.949_La_0.051_)_3_Ir_2_O_7_.

In order to obtain detailed information on the changes in the energies and the spectral weights of the optical excitations with electron doping, we analyzed *σ*_1_(*ω*) of (Sr_1−*x*_La_*x*_)_3_Ir_2_O_7_ at 10 K by using the Drude-Lorentz oscillator model^24^,^25^:


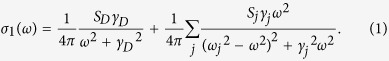


The first term represents the contribution from the Drude response. *S*_*D*_ and *γ*_*D*_ denote the strength and the scattering rate of the Drude peak. The second term corresponds to bound excitations. *S*_*j*_, *ω*_*j*_ and *γ*_*j*_ are the strength, resonance frequency, and the width of the *j*th Lorentz oscillator, respectively. The coherent response of (Sr_1−*x*_La_*x*_)_3_Ir_2_O_7_ cannot be explained in terms of a simple Drude oscillator, which is a typical characteristic of correlated electron materials. We used a combination of a Drude and a Lorentz oscillators to fit the coherent response located at *ω* < 0.05 eV. We stress that the accurate information on the bare electronic structure can be gained from the analysis of *σ*_1_(*ω*) at the lowest temperature 10 K. As shown in Fig. 2(a,b), the conductivity spectra at high temperatures are significantly different from the data at 10 K. The increase in temperature leads to the filling of the optical gap as well as the redshift of the peak *α*. This behavior was found not to be associated with the changes in the bare electronic structure but with the variation of the contribution from the thermal carriers^23^.

**Figure 3:**
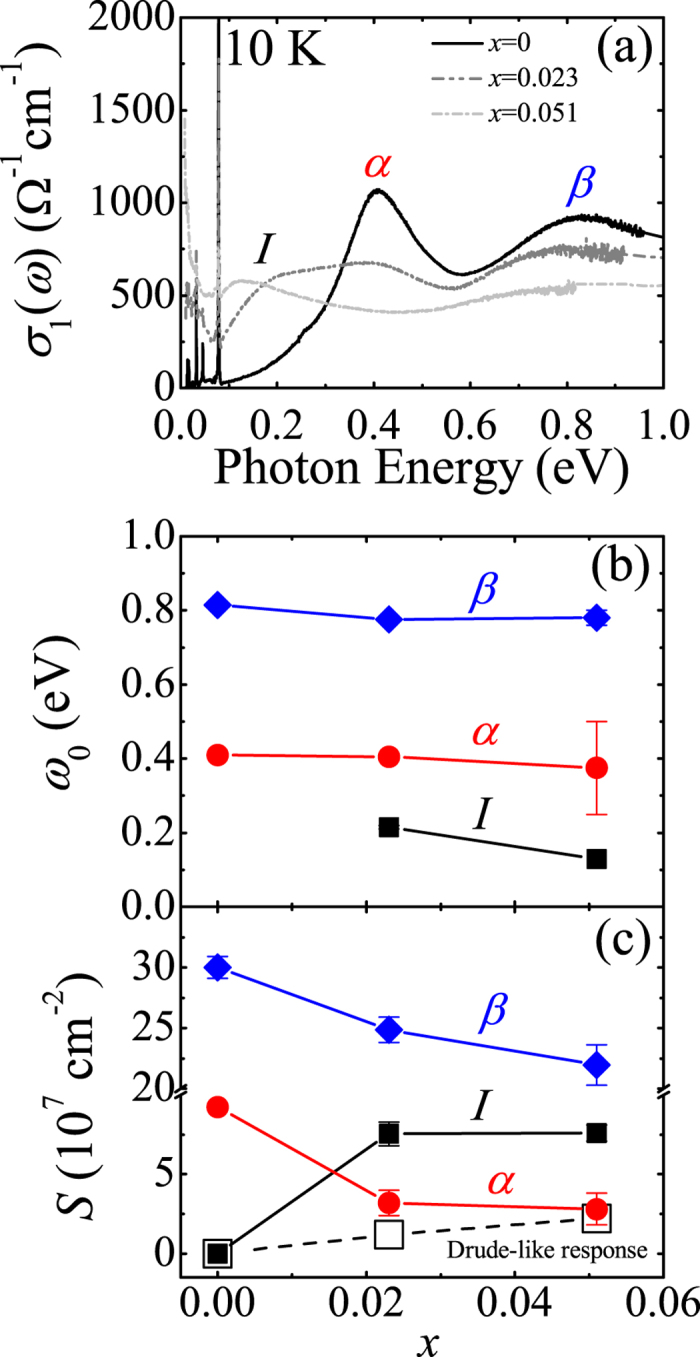
(**a**) *σ*_1_(*ω*) of (Sr_1−*x*_La_*x*_)_3_Ir_2_O_7_ at 10 K. (**b**) Resonant frequencies of the peaks *I* (solid squares), *α* (solid circles), and *β* (solid diamonds). (**c**) Strengths of the Drude-like response (open squares), the peaks *I, α*, and *β*.

Figure 3 summarizes the evolution of the electronic structure with electron doping. The comparison of *σ*_1_(*ω*) at 10 K in Fig. 3(a) clearly illustrates the changes in the electronic structure; the suppression of the peaks *α* and *β* and the filling of the gap with ingap excitations (peak *I*) and a Drude-like response. In (Sr_0.977_La_0.023_)_3_Ir_2_O_7_, the incoherent response dominates the far-infrared conductivity. Conversely a coherent Drude-like response appears in the lowest energy region (<0.05 eV) of *σ*_1_(*ω*) of (Sr_0.949_La_0.051_)_3_Ir_2_O_7_. From the Drude-Lorentz model analysis, we find that the spectral weight transfer relevant to the insulator-metal transition occurs in the energy region where the optical transitions between the *J*_eff_ states are the main contributors to *σ*_1_(*ω*). We also find that the energy values of the peaks *α* and *β* barely change with electron doping. This indicates that the Mott gap does not shift much in energy with doping. The peak *I* on the other hand exhibits sizeable redshift of about 0.08 eV and is likely to be merged into the coherent response at higher doping levels.

The evolution of *σ*_1_(*ω*) of (Sr_1−*x*_La_*x*_)_3_Ir_2_O_7_ bears significant similarities to the filling-controlled Mott transition in strongly correlated materials such as 3*d* and 4*d* transition metal oxides^1^,^26^. The universal characteristics of the filling-controlled Mott metal-insulator transition in the context of infrared data include the following attributes: (i) The Mott gap excitation, i.e., the peak *α*, does not shift in energy appreciably but its intensity is suppressed^26^,^27^,^28^. The energy region below the gap is filled up with states in the metallic side of the transition. (ii) The optical conductivity contains a sizeable incoherent component before a fully coherent electronic response develops with the merger between the coherent and the incoherent contributions^26^,^29^,^30^,^31^. The renormalization of the response from the itinerant carriers and the resulting development of an incoherent response is a fundamental behavior of the optical spectra of Mott transition systems. Our conductivity data shown in Figs 2 and 3 clearly display the features associated with the Mott transition and therefore suggest the crucial importance of electronic correlations in the (Sr_1−*x*_La_*x*_)_3_Ir_2_O_7_ system.

A hallmark of strong electronic correlations is a substantial enhancement of the effective mass of the itinerant carriers of metallic compounds near the boundary of the metal-insulator transition^1^,^26^,^32^. Having observed the spectroscopic signatures of the electronic correlations, we now estimate the mass enhancement to quantify the degree of the electronic correlations in (Sr_0.949_La_0.051_)_3_Ir_2_O_7_ by applying a sum rule to *σ*_1_(*ω*). As a dimensionless measure of spectral weight, we calculate the effective electron number *N*_eff_(*ω*)^26^ by using


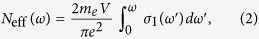


where *m*_*e*_ is the free electron mass and *V* is the volume containing one Ir^4+^ ion. If the high-frequency cutoff *ω*_c_ of the integral is chosen to include only the intraband contribution to *σ*_1_(*ω*), then


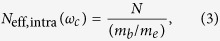


where *m*_*b*_ is a band mass and *N* is the number of itinerant carriers or band filling^26^,^33^. For correlated electron materials, the intraband response from the itinerant carriers is expected to separate into coherent and incoherent components due to many-body effects^1^,^26^. The former corresponds to the Drude-like mode centered at *ω* = 0 eV. If the high-frequency cutoff of the integral in equation (2) is chosen to contain the coherent component of the itinerant response, then


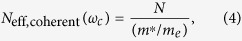


where m* is the effective mass^26^,^33^.

In metallic (Sr_0.949_La_0.051_)_3_Ir_2_O_7_, the nominal number of itinerant electrons *N* should be 3*x*/2 = 0.0765. The effective number of electrons responsible for the intraband conductivity *N*_eff,intra_ can be estimated from equation (3) with *ω*_c_ = 0.37 eV below which the Drude-like peak and ingap excitation are main contributors to the conductivity. The calculation gives *N*_eff,intra_ ≈ 0.057, thus indicating *m*_*b*_ ≈ 1.3*m*_*e*_. The effective electron number associated with the coherent component can be calculated by choosing the high-frequency cutoff of equation (4) as 0.055 eV below which the Drude-like mode dominates the conductivity. The value of *N*_eff,coherent_ is found to be about 0.014, yielding m* ≈ 5.5*m*_*e*_. Our sum rule analysis therefore indicates a mass enhancement of m* ≈ 4.2*m*_*b*_.

**Figure 4:**
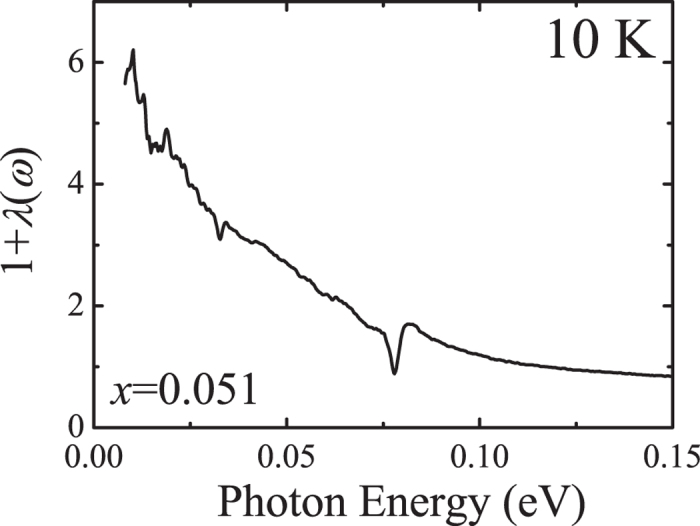
Mass enhancement of (Sr_0.949_La_0.051_)_3_Ir_2_O_7_ at 10 K calculated from the extended Drude model analysis.

An alternative method of estimating the mass enhancement is the extended Drude model analysis. The extended Drude model where interacting systems are mapped into the free electron model with an effective mass introduces frequency-dependent mass enhancement^26^,^34^


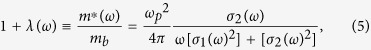


where *ω*_*p*_ is the plasma frequency of the intraband response and *σ*_2_(*ω*) (*σ*_1_(*ω*)) is an imaginary (real) part of the optical conductivity. The plasma frequency was calculated by using the relation 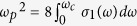 with the high-frequency cutoff of *ω*_c_ = 0.37 eV. As displayed in Fig. 4, the mass enhancement estimated from the extended Drude model analysis reaches about 6 at the lowest energy, which is close to the value from our sum rule analysis.

The mass enhancement of (Sr_1−*x*_La_*x*_)_3_Ir_2_O_7_ from our analyses is comparable to those of strongly correlated 3*d*/4*d* transition metal oxides. For the cuprates, the mass enhancement ranges from 2 to 10^30^,^35^,^36^. The mass enhancement of VO_2_ which is one of the representative examples of Mott system was about 5 near the metal-insulator transition^37^. The perovskite titanates and vanadates were found to have the mass enhancement of about 4–12^38^,^39^,^40^,^41^. A 4*d* itinerant ferromagnet SrRuO_3_ had the mass enhancement of about 6^42^. Large mass enhancement of (Sr_0.949_La_0.051_)_3_Ir_2_O_7_ can be due to the formation of the very narrow *J*_eff _= 1/2 bands near the Fermi level^2^,^4^. Polaronic or excitonic effects could also contribute the mass enhancement^26^,^43^,^44^,^45^,^46^,^47^. Previous spectroscopic experiments on Sr_2_IrO_4_ and Sr_3_Ir_2_O_7_ suggested an importance of electron-phonon coupling. The electron-phonon coupling can induce a renormalization of intraband response and lead to the formation of an incoherent side band in the optical conductivity. The polaronic side band should show strong temperature evolutions^26^,^43^. We note that the ingap excitation in *σ*_1_(*ω*) of (Sr_0.949_La_0.051_)_3_Ir_2_O_7_ displays negligible temperature dependence, indicating a minimal role of the electron-phonon coupling. Therefore our result indicates that the electronic correlations in (Sr_1−*x*_La_*x*_)_3_Ir_2_O_7_ are as strong as those in 3*d*/4*d* transition metal oxides and play a critical role in the charge dynamics of (Sr_1−*x*_La_*x*_)_3_Ir_2_O_7_.

## Conclusion

We investigated the electronic response of the (Sr_1−*x*_La_*x*_)_3_Ir_2_O_7_ system by using infrared spectroscopy. A filling-controlled insulator-metal transition revealed in our optical conductivity data bore striking similarities with those of strongly correlated electron matters such as 3*d*/4*d* transition metal oxides. The sum rule and the extended Drude model analyses further revealed a substantial mass enhancement of the itinerant carriers of the metallic compound near the metal-insulator transition boundary. Our experimental data obtained by using a bulk-sensitive probe and the model-independent analyses demonstrate a critical role of the electronic correlations in the (Sr_1−*x*_La_*x*_)_3_Ir_2_O_7_ system.

## Methods

Single-crystals of (Sr_1−*x*_La_*x*_)_3_Ir_2_O_7_ (*x* = 0, 0.023 and 0.051) were grown via flux techniques. Dopant content was determined by energy-dispersive x-ray spectroscopy measurements. Details of the growth procedure were described elsewhere^17^.

We measured the *ab*-plane reflectivity spectra *R*(*ω*) in the photon energy region between 5 meV and 1 eV using a Fourier transform infrared spectrometer (Bruker VERTEX 70 v). An *in-situ* gold overcoating technique^48^ was used to compensate the effect of rough sample surfaces. The dielectric constants, *ε* = *ε*_1_ + *iε*_2_, in the energy range from 0.74 to 5 eV were obtained by using spectroscopic ellipsometer (V-VASE, J. A. Woollam Co.). For the low-energy spectra below 5 meV, *R*(*ω*) was extrapolated by using the Hagen-Rubens relation^24^. We transformed *R*(*ω*) to obtain the complex conductivity *σ*(*ω*) = *σ*_1_(*ω*) + *iσ*_2_(*ω*) through the Kramers-Kronig analysis.

## Additional Information

**How to cite this article**: Ahn, G. *et al*. Infrared Spectroscopic Evidences of Strong Electronic Correlations in (Sr_1−*x*_La_*x*_)_3_Ir_2_O_7_. *Sci. Rep.* **6**, 32632; doi: 10.1038/srep32632 (2016).
